# Partial sleep deprivation with a 2-h time-in-bed may not change divergent thinking and insight problem solving in young males: a Bayesian analysis

**DOI:** 10.1186/s40101-026-00431-z

**Published:** 2026-05-21

**Authors:** Yuki Motomura, Toshihiro Iwayama, Tamaki Ueda, Mayu Kajihara, Takayuki Momoi

**Affiliations:** 1 Faculty of Design, Kyushu University, Fukuoka, Japan; 2 Graduate School of Integrative Frontier Science, Kyushu University, Fukuoka, Japan; 3 Railway Technical Research Institute (Current Affiliation), Tokyo, Japan

**Keywords:** Sleep deprivation, Creative cognition, Divergent thinking, Insight

## Abstract

**Background:**

This study investigated the impact of partial sleep deprivation on creativity by comparing performance changes between the partial sleep deprivation and normal sleep groups before and after engaging in creative tasks.

**Methods:**

Twenty-seven adult males (age 22.59 ± 1.89) participated in this experiment. Participants were divided into two groups: those with partial sleep deprivation and those with normal sleep patterns. Both groups completed creativity assessments, including the Japanese version of the Divergent Association Task (DAT) and tangram puzzles, before and after the sleep intervention.

**Results:**

The results indicated that despite increased subjective sleepiness and decreased sustained attention in the partial sleep deprivation group, there were no significant differences between the two groups in terms of performance on the DAT and tangram tasks. A Bayesian two-way analysis of variance supported the hypothesis that partial sleep deprivation does not adversely affect these aspects of creativity.

**Conclusions:**

These findings suggest that partial sleep deprivation may not impair certain creative abilities, such as verbal divergent thinking and insight problem-solving. However, while creative performance is maintained, other cognitive functions and overall health may be compromised owing to insufficient sleep. Therefore, individuals, especially those in creative professions, should be cautious of the potentially broader impacts of sleep deprivation.

## Background

Creative activities can lead to short sleep duration or irregular sleep patterns. King et al. investigated sleep among interior design students during project-based workshop sessions. It reported that 79% of students sleep less than 7 h at least three nights per week, and many students alternate between nights of sleep restriction (short sleep) and nights of recovery (long sleep). Considering that sleep deprivation affects many mental functions [1], creativity is expected to be no exception. However, details of the relationship between sleep and creativity remain unclear.

Creativity encompasses multiple abilities. This study adopted an approach that focused on a specific cognitive domain to scientifically capture the highly heterogeneous concept of creativity. Dietrich and Kanso [2] classify creativity into three components: insight, divergent thinking, and artistic creativity. Insight is considered a productive form of thinking that generates new solutions to problems rather than a reproductive form of thought that applies previously experienced solutions [3]. Divergent thinking is the ability to generate multiple solutions to problems without upper limits or definitive conclusions [4]. The quantitative assessment of artistic creativity is difficult in laboratory settings. Therefore, this study focuses on insight and divergent thinking, which have been established as objective indicators.

Sleep deprivation adversely affects cognitive and psychomotor performance. Dinges et al. [5] restricted the sleep duration of participants to 33% less than their habitual sleep time (resulting in an average of 4.95 h of sleep per night) and had them perform task-related exercises. Their results suggested that even a single night’s sleep restriction is associated with a decline in performance. Studies examining the relationship between sleep deprivation and brain function have suggested that the prefrontal cortex, which is involved in arousal, attention, and the ability to make and process appropriate judgments, is particularly vulnerable to sleep deprivation, thereby affecting goal-directed behaviors and sustained attention [6, 7]. Previous studies have shown that basic cognitive functions, such as executive function, attention, and working memory, form the foundation of creativity [8, 9]. Therefore, a decline in the cognitive abilities underlying creativity due to sleep deprivation is expected to lead to a decrease in creativity.

However, findings regarding the impact of creativity on sleep deprivation remain controversial. It has been suggested that one night of total sleep deprivation impairs the flexibility of divergent thinking [10]. Fluency, originality, and flexibility scores on the Language Form of the Torrance Tests of Creative Thinking have been shown to deteriorate following partial sleep deprivation [11]. A systematic review by Lim et al. [12] identified eight studies on creative task performance under sleep-deprived conditions. Overall, the review concluded that sleep deprivation tends to impair creative ability. Divergent linguistic thinking appears to be particularly more vulnerable. However, owing to the limited quality and small sample sizes of these studies, their conclusions are far from definitive. Moreover, all studies employed interventions based on total sleep deprivation (i.e., staying awake overnight).

Conversely, some reports have suggested that sleep deprivation may be beneficial for memory consolidation and for solving insight problems. Landmann et al. [13] investigated the effects of sleep on memory consolidation and reorganization and reported that sleep deprivation was beneficial for memory restructuring and solving insight problems. The participants were divided into groups based on their sleep or waking conditions (nighttime sleep, nighttime sleep deprivation, and daytime waking) and performed tasks before and after the respective conditions. The Compound Remote Associates Task, a creativity test in which participants are asked to find a word that can be linked to each of three seemingly unrelated words, was implemented. The nighttime sleep deprivation group showed significantly shorter response times to problems that were not solved during the first administration of the task than the nighttime sleep and daytime waking groups. Furthermore, Lacaux et al. [14] reported that drowsiness, the state between sleep and wakefulness, may promote creativity. In their experiment, participants solved mathematical insight problems before and after taking a 20‐min break in a dark room. During the break, participants who transitioned to non-REM stage N1 sleep were 2.7 times more likely to detect a hidden problem-solving strategy than those who did not fall asleep.

The relationship between sleep deprivation and creativity has been inconsistent across studies, and further investigation is needed. In everyday life, people rarely experience a full night of sleep deprivation but rather often accumulate sleep debt because of shortened sleep. Although Nelson et al. [11] utilized partial sleep deprivation in their study, their protocol involved only a 30-min nap during a 24-h wake period, which represents a relatively intense form of partial sleep deprivation. On the other hand, a study analyzing medical interns’ sleep during extended overnight duty using actigraphy reported that their sleep duration was approximately 2 h [15]. Whether creative performance is affected under 2-h partial sleep deprivation, which occasionally occurs during overwork conditions, remains unclear.

In this study, we focused on aspects within the definitions of insight and divergent thinking that pertain to generating new solutions to a given problem and developing novel ideas under specific conditions, to examine their relationship with partial sleep deprivation. The participants were divided into groups based on their sleep conditions (normal sleep 8 h; partial sleep deprivation 2 h), and creativity tasks were administered before and after sleep manipulation. The creativity tasks included a tangram task, an insight problem [16], and a Divergent Association Task (DAT) that assessed linguistic divergent thinking.

### Aims

This study investigated the effects of partial sleep deprivation on creative performance by focusing on verbal divergent thinking and insight problem-solving abilities. This study sought to clarify whether a single night of restricted sleep (2 h) significantly impairs these aspects of creativity compared with a full night of sleep (8 h).

The following hypotheses were tested:

1. Partial sleep deprivation leads to decreased DAT performance, indicating reduced verbal divergent thinking ability.
2. Partial sleep deprivation results in fewer correct and diverse solutions to tangram-based insight problems, reflecting reduced problem-solving flexibility.

## Method

### Ethics

All participants provided informed consent and the study was conducted in accordance with the Declaration of Helsinki. The study protocol was approved by the Ethics Committee of the Faculty of Design at Kyushu University (approval number 373). This study was conducted from October 2020 to October 2021.

### Preliminary screening for participant selection

The purpose of the preliminary screening was to ensure balanced creative abilities, sleep characteristics, and mood states between the two groups (sleep and partial sleep deprivation groups) in this experiment. Participants were selected using the tangram task and a sleep questionnaire. The DAT was not included in the preliminary screening because it reduced the number of available word sets. The inclusion criteria were as follows: males aged 18–30 years, no current or past mental illness or sleep disorder, no medications affecting sleep, and the ability to adhere to the study schedule.

The study was conducted between October 2020 and October 2021. A total of 46 male participants (mean age 21.8 ± 2.0 years, range 19–26 years) from the Faculty of Design at Kyushu University took part in the preliminary survey. Prior to the experiment, participants were provided with explanations of the experimental procedures and important considerations. Consent for participation was obtained via an online program (because the experiment was conducted during the COVID-19 pandemic). The eligibility criteria were that the participants had no current or past psychiatric or sleep disorders, were not taking any medications that affected their sleep, and were able to adhere to the study schedule. Participants self-declared that they met the inclusion criteria.

Before the experiment, an email containing a link to the online program and tangram task was sent to each participant. The deadline for completing the task was set within 2 weeks of receiving the email.

Prior to the experiment, the participants completed questionnaires regarding their sleep habits and mental health through an online program. On the day of the experiment, between 10:00 a.m. and 12:00 a.m., the participants answered a questionnaire on sleepiness via an online program at home and completed the tangram task. After completing the task, the participants were asked to send the results of the creative task via email.

### Questionnaires on preliminary survey

The Pittsburgh Sleep Quality Index (PSQI) is a questionnaire designed to measure sleep health by assessing items such as sleep quality, sleep onset latency, sleep duration, sleep efficiency, sleep disturbances, the use of sleeping medications, and daytime dysfunction [17]. The participants were asked to complete the PSQI between receiving the URL for the online program and performing the tangram task.

The K6 is a screening tool for depression and anxiety disorders. Its optimal cutoff score is 13, with a score of 13 or higher indicating potential depression or anxiety disorder [18]. The participants were required to complete the K6 during the period between receiving the URL for the online program and undertaking the tangram task.

The results of this questionnaire were collected for use in the subsequent main experiment.

### Tangram task for the preliminary survey

In this study, a tangram task, a type of insight problem, was employed as a creativity task. A tangram is a mathematical puzzle in which seven pieces (triangles and quadrilaterals cut from a square) are arranged to form various shapes. This has been used in previous studies as a measure of insight [16].

**Fig. 1:**
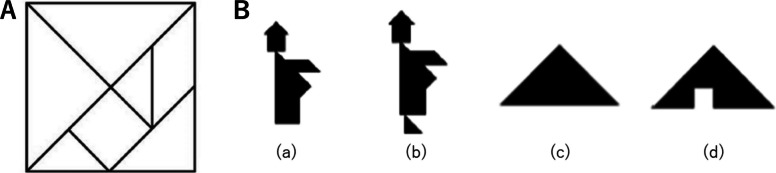
Tangram task for the preliminary survey. Tangram materials and sample tasks used in the preliminary survey. **A** Standard set of seven geometric pieces derived from a square, used for all tangram tasks. **B** a–d represent the specific tangram problems presented to participants during the preliminary phase, which required them to reconstruct each shape using all seven pieces. These tasks were used to assess baseline insight problem-solving ability

The participants were sent an email with an attached file containing the image shown in Fig. 1A and were instructed to print the file before the experiment and cut the figure into seven pieces. During the task, the participants were instructed to use all seven pieces shown in Fig. 1A to create each tangram, as shown in Fig. 1B (a total of four tangrams). A time limit of 600 s (10 min) was set for each tangram and the time taken to complete each tangram was recorded. All participants were instructed to perform the task under the same conditions (solving the tangrams in the order of (a), (b), (c), and (d), as shown in Fig. 1B, and to work continuously without taking breaks or sleeping. Participants were asked to record a photograph of their completed tangram and the time taken to complete it after finishing the task and to submit them via email to the conductor of the experiment.

The task of the main experiment was to examine the number of different combinations that could be created from a single tangram within the time limit. To reduce learning effects, a task different from the tangram task used in the main experiment was administered during the preliminary survey; however, it was designed such that the participants could approach the tangram in a similar manner.

## Main experiment

### Participants

The PSQI and K6 scores obtained from the preliminary survey were used to screen participants for the main experiment. For participants whose interval between the preliminary survey and the main experiment exceeded 1 month, the PSQI and K6 were administered again. Furthermore, to equalize the participants’ ability to solve the tangram task, individuals were sequentially assigned to two groups (sleep and partial sleep deprivation), starting with those whose average tangram rank from the preliminary survey was the closest to the overall mean. Participants were ordered by preliminary tangram performance (closest to the overall mean first) and then sequentially allocated to the normal sleep or PSD group to minimize between-group imbalances in age, PSQI, K6, and major.

A power analysis was conducted using G*Power (https://www.psychologie.hhu.de/arbeitsgruppen/allgemeine-psychologie-und-arbeitspsychologie/gpower). The interaction term in a two-way ANOVA was then analyzed. Calculations were performed with an effect size of Cohen’s f = 0.25 (moderate), α = 0.05, and 1-β = 0.8. Based on these parameters, the target sample size was determined to be 28 participants.

After selecting a target sample size of 28 participants, one participant withdrew. Consequently, the experiment was ultimately conducted with 27 participants (22.6 ± 1.9 years old). Ultimately, 27 participants volunteered and were divided into two groups, such that variations in age, tangram-solving ability (measured by the average rank in the preliminary survey), PSQI scores, and K6 scores were balanced. Additionally, efforts were made to distribute university majors (e.g., ergonomics, industrial design, acoustic design, art, and information design) as evenly as possible between the groups. The normal sleep group comprised 13 participants (22.4 ± 1.8 years old), and the partial sleep deprivation group comprised 14 participants (22.8 ± 1.9 years old). The average tangram rank was 18.2 ± 3.2 for the normal sleep group and 17.6 ± 3.7 for the partial sleep deprivation group, and the average K6 scores were 5.3 ± 2.7 and 4.9 ± 2.9, respectively.

### Experimental procedure

For the 2 days preceding the experiment, the participants were instructed to sleep for 8 h, with their bedtime set 4 h before and their wake-up time 4 h after the individual sleep midpoint, as determined by the PSQI. Additionally, for any participant whose bedtime would otherwise fall later than 1:00 a.m., sleep was regulated so that they went to bed at 1:00 a.m. and woke up at 9:00 a.m. This was applied to seven participants in the normal sleep group and three in the partial sleep deprivation group. Moreover, during the 2-day sleep regulation period prior to the experiment, participants were prohibited from taking naps and heavy drinking.

On the day before the experiment, all participants underwent a polymerase chain reaction (PCR) test as a preventive measure against COVID-19. Only the participants who tested negative on the first day of the experiment were included.

**Fig. 2:**
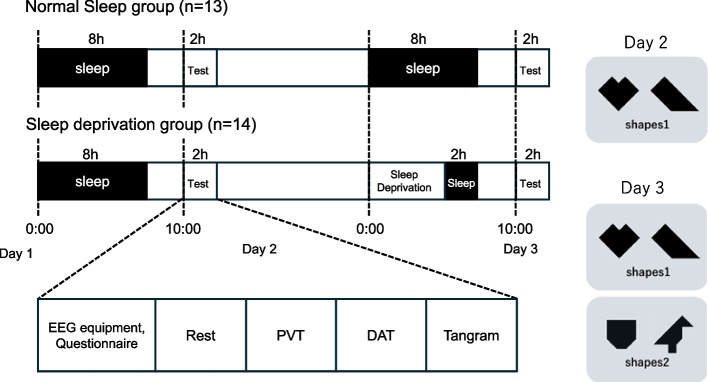
Experimental protocol. **A** The timeline of the 3-day experimental protocol for the normal sleep (*n* = 13) and partial sleep deprivation (*n* = 14) groups. The participants completed a sequence of tasks each morning, including filling the questionnaire, the EEG setup, eyes-open resting state, the Psychomotor Vigilance Task (PVT), the Divergent Association Task (DAT), and the tangram-based insight tasks. **B** Tangram task conditions: on day 2, the participants solved two silhouettes from “shapes1” (Heart and Square), and on day 3, they either repeated the same two shapes (shapes1) to assess the incubation effects or solved for two new shapes (shapes2: Pot and Bird) to examine transfer and generalization

The experiment was conducted over three consecutive days (Fig. 2A). During the experimental period, participants were not permitted to consume tobacco, caffeine, or alcohol, or engage in vigorous exercise, exciting activities, puzzle games, or napping. However, to minimize stress, activities that did not fall under these prohibitions, such as working on class assignments, reading, playing games without puzzles, using the internet, or conversing with experimenters or other participants, were allowed at the discretion of the test conductors. During the experiment, if a participant appeared likely to fall asleep, the test conductor spoke to them to maintain their wakefulness. During non-task periods, the participants stayed in the Human Factors Laboratory on the fourth floor of Building 1 at Kyushu University’s Ohashi Campus, where the room temperature was maintained at 26 ± 2 °C. Although the basic set temperature was 26 °C, the room temperature was increased if any participant complained of feeling cold. To control for the effects of dietary metabolism and blood sugar fluctuations on cognitive function, all meals during the experiment period were prepared by the test conductor. The same menu was provided to all participants simultaneously (breakfast: bread, yogurt, etc.; lunch: curry, etc.; dinner: bento box, etc.).

On the first day of the experiment, the participants were required to arrive at the experimental room at least 1 h before their scheduled bedtime during the sleep regulation period. In the experimental room, after being fitted with the wearable device Fitbit Inspire2 (Fitbit, Inc.), the participants were instructed to sleep for 8 h under the same bedtime and wake-up times as those established during the sleep regulation period.

On the second day, after waking up, washing their faces, brushing their teeth, and finishing breakfast, 1.5 h after their wake-up time, electrodes for electroencephalography (EEG) were attached, and the participants completed a series of tasks: an eyes-open resting period, the Psychomotor Vigilance Test (PVT), the DAT, and the tangram task (using shapes labeled Heart and Square [shapes1]). EEG measurements were conducted in a soundproof, radiofrequency-shielded room on the fourth floor of Building 1. During the task performance, the participants wore short-sleeved shirts and shorts. The room conditions were strictly controlled: temperature at 26 °C, humidity at 50%, and desk illuminance between 550 and 650 lx. The electrode cap was removed after measurement. On the night of the second day, after donning the wearable device, the participants were allowed either 8 h (normal sleep group) or 2 h (partial sleep deprivation group) of sleep, and the partial sleep deprivation group was required to go to bed 2 h before their scheduled wake-up time.

On the third day, after waking up, washing their faces, brushing their teeth, and finishing breakfast, 1.5 h after the wake-up time, EEG electrodes were once again attached, and the participants performed the following tasks: an eyes-open resting period, the PVT, the DAT, and the tangram task (using shapes labeled as Heart and Square [shapes1] and Bird and Pot [shapes2]) (Fig. 2B). On each experimental day, a maximum of two participants were tested simultaneously. In cases where two participants were scheduled concurrently, the second participant’s session commenced immediately after the first participant completed their session; consequently, the start time for the second participant was approximately 2.5–3 h after the wake-up time. In total, 15 participants (7 in the normal sleep group and 8 in the partial sleep deprivation group) participated in the first session and 12 participants (6 in each group) participated in the second session. However, the EEG results are not reported here.

### Objective sleep duration

Fitbit's algorithm uses information on body movement and pulse waves during sleep to estimate the total sleep duration and the duration of light sleep, deep sleep, and REM sleep. The sleep vs. wakefulness classification has been shown to agree with polysomnography (PSG) classifications 76% of the time [19]. Sleep from the night of experiment day 2 was analyzed to verify the sleep intervention effects in each group. Sleep stage classification could not be applied to the 2-h sleep period in the partial sleep deprivation group in this study, and only the total sleep duration was analyzed.

### Questionnaires

The State-Trait Anxiety Inventory (STAI) [20] is a 20-item questionnaire designed to measure state anxiety. To assess subjective sleepiness and mood, participants were instructed to complete the SSS and STAI via an online program immediately after starting the experimental session.

### Psychomotor vigilance task

The PVT was administered to objectively assess sleepiness. It measures sustained attention and is an objective indicator of decreased alertness [21]. In this study, a 5-min PVT was conducted on a tablet device (FFF SMART LIFE CONNECTED, FFF-TAB7) using the open-source software Vigilance Buddy 1.53 (https://researchbuddies.com/). In this task, a stimulus displayed as a video of incrementing numbers was presented on the screen and the participants were instructed to tap the screen as quickly as possible upon noticing the stimulus. Reaction time from the onset of the stimulus to the screen tap was measured. The primary outcome measure analyzed was the reciprocal of the mean reaction time, with a lower value indicating a decline in sustained attention [22].

### Creativity tasks

#### Divergent association task

The DAT was used to measure linguistic divergent thinking. It requires participants to list 10 nouns that are as different as possible in terms of meaning or use within a four-minute period [23]. It is possible to objectively quantify divergent thinking abilities by calculating the semantic distances between these nouns. DAT performance is correlated with other divergent thinking tasks (e.g., the alternative use task). We used the DAT because it provides brief administration and objective, automated scoring based on semantic distance, addressing limitations of traditional divergent thinking tasks such as the AUT that require labor-intensive and sample-dependent scoring.

In this study, a Japanese version of the DAT was developed based on an algorithm published by Olson et al. [23] (https://osf.io/bm5fd/) and was employed as a task. The DAT scores were calculated using Google Colaboratory as the Jupyter notebook execution environment, and a pre-trained Japanese word vector file (.gz) was obtained online (https://fasttext.cc/docs/en/crawl-vectors.html). Following the scoring method of the English version, the first seven valid responses out of the 10 nouns provided by the participants were used to compute 21 pairwise semantic distances. The DAT score is defined as 100 times the average semantic distance. The DAT score was defined as 100 × the mean pairwise semantic distance; the theoretical range is 0–200, while scores commonly range around 65–90 and rarely exceed 100 in practice. Notably, a significant correlation was observed (*r* = 0.50, *p* < 0.001) between the “scores calculated by the Japanese-language algorithm” and the “scores calculated by the English-language (original) algorithm” using the English translations of the Japanese response data obtained in this experiment. This confirms that the Japanese-language DAT score is a useful indicator of creativity.

For this task, the participants were given an A4 sheet that included task instructions, precautions, and an answer section, and were asked to write their responses using a ballpoint pen. Although the time limit for the task was four minutes, the task was concluded once the participant recorded 10 nouns. Furthermore, as the DAT was administered twice (either before and after sleep or before and after partial sleep deprivation) on the third day, the participants were required to provide responses using a different set of nouns from those used on the second day.

### Tangram task

The tangram task was used as a creativity test to measure insight. The tangrams used in the second experiment were modeled using the 3D CAD software “Fusion 360” (.stl files). From these models, the 3D printer files (.gx files) were created using the slicing software “FlashPrint.” Subsequently, using a 3D printer (“FLASHFORGE Adventurer3”), 22 sets of squares—each composed of 7 pieces and measuring 100 mm (height) × 100 mm (width) × 10 mm (thickness)—were produced.

In the tangram task, participants were required to determine the number of different combinations that could be created for a given silhouette. Each silhouette was allotted a time limit of 7 min. Two types of comparisons were performed for the tangram task: Research Question 1 (RQ1), in which participants solved the same silhouette on different days to investigate the incubation effect, and Research Question 2 (RQ2), in which participants solved a new silhouette.

RQ1 involved solving the same silhouette (Shape 1) on both the second and third days to verify the incubation effect. The incubation effect refers to the phenomenon in which temporarily interrupting task-solving and inserting a rest period (or another task or sleep) increases subsequent solution rates and insights (sudden realizations). In classical models of the creative process, this is positioned as the second stage: “Preparation → Incubation → Insight → Verification.” Meta-analyses show the effect size varies depending on the presence/length of the break and the task type (with a tendency for greater effect in divergent tasks). Additionally, a mechanism called “fixation forgetting” has been proposed, suggesting that fixation on initial erroneous representations weakens during the break, relatively restoring access to the correct solution [24]. This study treated retesting the same silhouette (day 2 → day 3) as “reconstruction after a break” and examined the potential contribution of incubation. In RQ2, on the second and third days, the participants solved the same silhouette (shapes1) to examine the incubation effect. In addition, by having the participants solve shapes1 on the second day and a different silhouette (shapes2) on the third day, the impact of different sleep conditions on their responses to a new creativity task was investigated.

The experiment used two silhouettes on the second day (Heart and Square: shapes1); on the third day, we used the same silhouettes (Heart and Square: shapes1) and two new silhouettes (Bird and Pot: shapes2). When only a single silhouette is used, the individual strengths and weaknesses (task-specific variance) of that particular shape can strongly influence the results. Therefore, we expected to obtain a more stable indicator of creativity by averaging the scores from two different silhouettes to level out task-specific variance. Given that the Heart and Pot shapes are bilaterally symmetrical, creating one combination might inadvertently lead to the formation of a symmetrical duplicate. Therefore, participants were instructed not to produce bilaterally symmetrical combinations.

To account for order effects, the experiment was conducted in four different orders: two orders on the second day (Square → Heart or Heart → Square) and two orders on the third day (Bird → Heart → Pot → Square or Square → Pot → Heart → Bird), with counterbalancing among the participants.

### Statistical analysis

Statistical analyses were performed using JASP version 0.19.3.

Classical statistical hypothesis testing based on *p*-values alone cannot adequately support the hypothesis that there is no difference between groups. Therefore, a combined approach was employed that reported both Bayesian hypothesis testing using Bayes factors (BFs), as recommended by Keysers et al. [25], and conventional *p*-values. Bayesian hypothesis testing provides the strength of evidence for the null hypothesis relative to the alternative hypothesis, allowing for the statistical support of the absence of differences between groups. First, to determine whether there were differences in the demographic data between the groups, we conducted independent t-tests and Bayesian hypothesis tests on the K6 scores, tangram reaction order, and age from the preliminary survey.

In this study, creativity tasks and questionnaires were administered before and after sleep in both sleep and partial sleep deprivation groups. If the changes from pre- to post-sleep differed between groups, it was regarded as evidence of a sleep effect. Accordingly, statistical methods were used to test for interactions between the groups (two levels: normal sleep group vs. partial sleep deprivation group) and dates (two levels: day 2 vs. day 3).

Regarding the tangram tasks, two analyses corresponding to RQ1 and RQ2 were conducted.

RQ1: Verification of performance changes within the same task (i.e., comparing performance on the same tangram tasks—heart shape and square shape—on day 2 and day 3).

RQ2: Verification of performance generalization to different tasks (i.e., comparing performance across different tangram tasks: Heart and Square on day 2, Bird and Pot on day 3).

For the DAT task, since it was evaluated using a single analytical framework measuring the same “divergent thinking ability,” it was analyzed using the same method as the other tasks.

For each analysis, the mean number of responses was used as the outcome measure. For the STAI, SSS, PVT, DAT, and the number of responses in the tangram tasks with the same silhouette and those with different silhouettes, a mixed-design two-way analysis of variance (ANOVA) (group [two levels: normal sleep group, partial sleep deprivation group] × date [two levels: day 2, day 3]) was conducted. Both BFs and *p*-values were calculated. When the assumption of sphericity was violated, the Greenhouse–Geisser correction was applied to adjust the degrees of freedom.

For multiple comparison adjustments, post-hoc tests were performed using two-tailed t-tests with Holm-corrected *p*-values. For sleep duration on the second night of the experiment, unpaired t-tests were conducted between groups.

Participants with missing data for any analysis variable were excluded from the analysis of that specific variable. For objective sleep time data from the Fitbit, one participant from the normal sleep group and three participants from the sleep deprivation group were excluded. For the SSS data, one participant from the sleep deprivation group was excluded. For PVT data, one participant from the normal sleep group was excluded. For tangram data, one participant from the normal sleep group was excluded.

In this study, following Jeffreys’ [26] criteria, BF was interpreted as follows: a BF between 1 and 3 indicates little anecdotal evidence in favor of the alternative hypothesis. A BF value between 3 and 10 indicates moderate support for the alternative hypothesis. A BF of 10 or greater indicates strong support for the alternative hypothesis. Conversely, a BF of less than one indicates support for the null hypothesis. Specifically, a BF between 0.1 and approximately 0.333 indicates moderate support for the null hypothesis, and a BF of 0.1 or below is interpreted as strong evidence in favor of the null hypothesis. These criteria were used to evaluate the strength of evidence for the hypotheses in each statistical test.

## Results

### Demographic data

The demographic data are presented in Table 1. A Bayesian t-test was conducted to compare age, K6 scores, PSQI scores, and average tangram rankings between the sleep and partial sleep deprivation groups. The resulting BFs ranged from 0.358 to 0.4, providing modest support for the hypothesis that there were no differences between the groups (age: BF = 0.4; K6: BF = 0.38; PSQI: BF = 0.358; and tangram: BF = 0.382).

**Table 1:** Demographic data

	Normal Sleep	Sleep Deprivation	t	p	BF
Age	22.385 ± 1.968	22.786 ± 1.85	−0.545	0.591	0.4
Rank of tangram task	18.179 ± 3.874	17.607 ± 3.279	0.413	0.683	0.38
K6 (psychological distress)	5.308 ± 2.983	4.857 ± 2.84	0.401	0.692	0.358
PSQI (quality of sleep)	5.077 ± 2.303	5.071 ± 1.706	0.007	0.994	0.382
Sleep Duration (Day2 night)	378.667 ± 68.729	113.545 ± 11.945	8.471	< 0.001	1.61*10^8^

*Abbreviations*: *SD* standard deviation, *PSQI* Pittsburgh sleep quality index, *BF* Bayes factor

### State anxiety

**Fig. 3:**
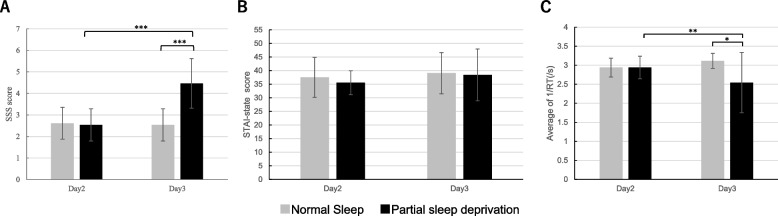
Subjective mood and subjective or objective sleepiness. Subjective and objective sleepiness and state anxiety scores across the two experimental days. **A** Stanford Sleepiness Scale score. On day 3, the partial sleep deprivation group reported significantly higher subjective sleepiness than the normal sleep group after the intervention (***p <.001). **B** State Anxiety Index. No significant differences were observed between groups or across days. **C** Reciprocal mean reaction time (1/s) for the Psychomotor Vigilance Task. A significant interaction was observed; the normal sleep group showed no performance change, while the partial sleep deprivation group showed a decline in performance on day 3 (*$p$ <.05, **$p$ <.01). Error bars represent the standard deviation

For the STAI results, a two-way ANOVA (group [two levels] × date [two levels]) was performed (Fig. 3A). The analysis revealed no significant main effects of group (F(1, 25) = 0.28, *p* = 0.600) or date (F(1, 25) = 1.71, *p* = 0.202). Moreover, no significant group × date interaction was observed (F(1, 25) = 0.15, *p* = 0.700). The Bayesian two-way ANOVA results supported the hypothesis of no group effect (BF = 0.314), provided modest support for the hypothesis of no time effect (BF = 0.434), and strongly supported the hypothesis of no group × time interaction (BF = 0.163).

### Subjective sleepiness

A two-way ANOVA (group [two levels] × date [two levels]) was conducted on the SSS results (Fig. 3B). The analysis revealed a significant main effect of group (F(1, 24) = 14.9, *p* < 0.001), with the partial sleep deprivation group exhibiting higher SSS scores than the normal. A significant main effect of date was also observed (F(1, 24) = 12.7, *p* = 0.002), with SSS scores being higher on day 3 than on day 2. Furthermore, a significant group × date interaction was observed (F(1, 24) = 14.9, *p* < 0.001). Post-hoc tests revealed that the SSS score on day 3 was significantly higher in the partial sleep deprivation group than in the normal sleep group (t(24) = − 4.856, *p* < 0.001), and that the SSS score on day 3 was significantly higher in the partial sleep deprivation group than on day 2 (t(12) = − 5.251, *p* < 0.001). The results of the Bayesian two-way ANOVA strongly supported the hypothesis of a group effect (BF = 955.978), a time effect (BF = 3673.978), and a group × time interaction (BF = 841.995).

### Objective sleepiness

For the PVT results, a two-way ANOVA (group [two levels] × date [two levels]) was conducted (Fig. 3C). Although there was a trend toward a group effect (F(1, 24) = 2.94, *p* = 0.099), no significant main effect of date was observed (F(1, 24) = 1.44, *p* = 0.242). However, a significant group × date interaction was observed (F(1, 24) = 9.75, *p* = 0.005). Post-hoc tests revealed that on day 3, the inverse of the mean reaction time was significantly lower in the partial sleep deprivation group than in the normal sleep group (t(24) = 2.344, *p* = 0.028). This suggests a decline in sustained attention. Furthermore, the partial sleep deprivation group showed a significantly lower inverse mean reaction time on day 3 than on day 2 (t(12) = 3.180, *p* = 0.004). The Bayesian two-way ANOVA results supported the hypotheses of a group effect (BF = 3.357) and group × time interaction (BF = 7.128) but provided inconclusive evidence of a time effect (BF = 2.331).

### Divergent association task

**Fig. 4:**
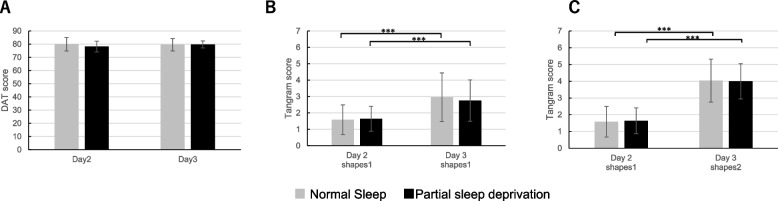
Creativity tasks. Creativity task performance across two experimental days. **A** Scores on the Divergent Association Task (DAT). No significant differences were observed between the sleep group and the partial sleep deprivation group across days. **B** Mean number of completions for repeated tangram shapes (shapes1) on day 2 and day 3. Performance improved on day 3 for both groups, suggesting a learning or incubation effect, but no group differences were found. **C** Mean number of completions for novel tangram shapes (shapes2) on day 3. Both groups generated more completions compared to day 2 shapes1, with no significant group differences, suggesting possible generalization of creative strategies. Error bars represent standard deviations

A two-way ANOVA (group [two levels] × date [two levels]) was conducted on the DAT results (Fig. 4A). The analysis revealed no significant main effects of group (F(1, 25) = 0.26, *p* = 0.611) or date (F(1, 25) = 0.46, *p* = 0.502). Furthermore, no significant group × date interaction was observed (F(1, 25) = 1.50, *p* = 0.231). The Bayesian two-way ANOVA results provided modest support for the hypotheses of no group effect (BF = 0.350), no time effect (BF = 0.266), and no group × time interaction (BF = 0.197).

### Tangram task (same shapes on days 2 and 3)

For the tangram task results using the same silhouette on both days (Fig. 4B), a two-way ANOVA (group [two levels] × date [two levels]) was performed. The analysis showed no significant main effect of group (F(1, 24) = 0.04, *p* = 0.849); however, a significant main effect of date was observed (F(1, 24) = 23.2, *p* < 0.001). No significant group × date interaction was observed (F(1, 24) = 0.27, *p* = 0.608). The Bayesian two-way ANOVA results provided marginal support for the hypothesis of no group effect (BF = 0.334), very strong support for the hypothesis of a time effect (BF = 745.720), and inconclusive evidence regarding the group × time interaction (BF = 0.406).

### Tangram task (different shapes on days 2 and 3)

A two-way ANOVA (group [two levels] × date [two levels]) was conducted on the tangram task results using different silhouettes on days 2 and 3 (Fig. 4C). The analysis revealed no significant main effect of group (F(1, 24) = 7.00 × 10⁻4, *p* = 0.979) and a significant main effect of date (F(1, 24) = 96.0, *p* < 0.001). No significant group × date interaction was observed (F(1, 24) = 0.04, *p* = 0.839). The Bayesian two-way ANOVA results provided modest support for the hypothesis of no group effect (BF = 0.352), very strong support for the hypothesis of a time effect (BF = 8.905 × 10⁹), and marginal support for the hypothesis of no group × time interaction (BF = 0.382).

## Discussion

In this study, we examined the effects of sleep deprivation on creativity by administering creativity tasks before and after a partial sleep deprivation simulation trial. Furthermore, we compared changes in performance between the partial sleep deprivation and normal sleep groups. The following discussion considers these findings.

First, there were no significant differences between the sleep and partial sleep deprivation groups in terms of demographic variables, questionnaire measures, or baseline tangram ability, indicating that the groups were well-matched. Second, no main effects or interactions were observed for state anxiety. In contrast, both SSS and PVT showed significant interactions: subjective sleepiness increased and sustained attention decreased on day 3 in the partial sleep deprivation group. These results indicate that the sleep manipulation effectively increased sleepiness and reduced vigilance. Although previous studies have reported that multiple days of partial sleep deprivation are associated with increased STAI scores [27, 28], the use of a single day of partial sleep deprivation, which is a milder intervention, may account for this discrepancy.

Conversely, the SSS showed a significant group × date interaction, with the partial sleep deprivation group exhibiting a marked increase in subjective sleepiness relative to the normal sleep group. In addition, the PVT results revealed a significant group × date interaction. Specifically, the partial sleep deprivation group demonstrated a decline in sustained attention following the intervention, as indicated by a lower reciprocal of the mean reaction time compared with the normal sleep group. These findings suggest that, although sleep manipulation did not alter state anxiety, it effectively increased the subjective and objective measures of sleepiness.

To examine the effect of partial sleep deprivation on linguistic divergent thinking, we investigated whether there was a group × time interaction in DAT performance. Regarding the DAT results, no significant main effects of group or time were observed and there was no significant group × time interaction. The Bayesian two-way ANOVA supported the hypothesis that there was no interaction. These findings suggest that neither short sleep duration (2 h) nor normal sleep (8 h) differentially affects linguistic divergent thinking. This is the first study to demonstrate the impact of partial sleep deprivation (with a 2-h time-in-bed) on creativity, which can be experienced in overwork conditions. A review of previous studies reported that compared with performance on visuospatial tasks, linguistic divergent thinking is more adversely affected by sleep deprivation [12]; therefore, the absence of a difference in our study was unexpected. This discrepancy may be due to differences in the forms of sleep deprivation. All the reviewed studies considered total sleep deprivation (i.e., staying awake overnight). Although partial sleep deprivation tends to preserve deep and rapid eye movement (REM) sleep, both of which are considered important for recovery because of sleep homeostasis [29], total sleep deprivation results in the loss of all sleep stages [30]. Since polysomnography was not performed in this study, it is speculative regarding sleep stages; however, maintaining deep sleep and REM sleep may have contributed to the recovery of divergent thinking. Although the intervention in this study was relatively milder than that in previous research, both subjective sleepiness and objective measures of sustained attention deteriorated significantly; however, creative performance was maintained despite a decline in other aspects of cognitive functioning.

Finally, we discuss the results of the tangram task. The Bayesian two-way ANOVA provided modest support for the hypothesis of no group effect, strong support for a time effect, and inconclusive evidence regarding a group × time interaction. These results indicate that it is difficult to attribute differences in the ability to repeatedly solve the same tangram task to the effects of sleep and partial sleep deprivation. However, the significant main effect of time, strongly supported by the Bayesian analysis, suggests that performance improved when the same tangram task was repeated after sleep or partial sleep deprivation. This improvement is likely attributable to the incubation effect resulting from performing the same task twice with an interval. Sleep deprivation shortens response times to problems that have previously been solved [13]. Thus, the incubation effect may have been offset by the detrimental effects of partial sleep deprivation on overall performance, leading to a lack of detectable group differences. However, the current results do not allow a detailed examination of this possibility.

Regarding Research Question 2, similar to Research Question 1, when comparing the performance of different tangram tasks between days 2 and 3, no significant group × time interaction or main effect of group was observed; however, a significant main effect of time was evident. The Bayesian two-way ANOVA provided modest support for the hypothesis of no group effect, strong support for a time effect, and modest support for a group × time interaction. These findings also suggest the generalization of the incubation effect, with the improvement being generalized to different tangram tasks. In addition, the results provided modest support for the hypothesis that partial sleep deprivation does not influence this change. It is conceivable that participants were better able to combine elements from their memory of the shapes solved on day 2 (shapes1) when tackling the same type of task (shapes2) on day 3, leading to an increased number of combinations. Alternatively, differences in the task difficulty may have contributed to this finding. The difficulty of the “shapes2” task could have been inherently lower than that of the “shapes1” task for the participants, irrespective of any incubation or sleep effects.

### Limitations

This study has several limitations. First, although creativity tasks were administered twice–before and after sleep–it was not possible to completely eliminate the learning effects or control for task difficulty across the days. Although these two tasks were designed to measure the same aspects of creativity, it is inherently difficult to create tasks that are both difficult and free from learning effects when measuring creative performance.

Second, in the tangram task, it was difficult to objectively determine whether the participants experienced a fixation or impasse, which is a key component of insight problem-solving. If the participants had not experienced an impasse, the task might not have functioned effectively as an insight problem. However, if the task difficulty increased significantly, most participants were likely to remain stuck and unable to find solutions. This highlights the importance of carefully adjusting the task difficulty when using insight problems to measure creativity in experimental settings. Furthermore, whether one experiences an impasse in a particular insight problem depends heavily on individual traits or prior experiences, making it particularly challenging to use insight-based tasks uniformly across participants.

Because the present PSD manipulation (2-h TIB) is more severe than common daily-life restriction, generalization to milder chronic restriction should be made with caution. A control condition involving a waking interval was necessary to separate the effects of learning from those of sleeping. However, implementing a waking condition of equivalent length would result in total sleep deprivation, making it difficult to isolate the learning effects. Another limitation is that the DAT used was a Japanese version for which translation and cross-linguistic validity have not been formally verified. Finally, the generalizability of the results is limited because the study included only male participants. The primary reasons for including only males in this study were experimental constraints and the need to control for physiological confounders. First, in female participants, the menstrual cycle could potentially affect sleep quality, mood, and cognitive abilities, making strict control difficult. Second, the experiment was conducted during the COVID-19 pandemic, resulting in extremely limited personnel, time, and facility resources (e.g., separate changing areas and female staff). This necessitated the restriction of participation to male participants. Future studies should include female participants.

## Conclusion

Despite the partial sleep deprivation group reporting greater subjective sleepiness and showing a decline in sustained attention compared to the normal sleep group, no group differences were observed in the linguistic divergent thinking task. Although the BF did not meet Jeffreys’ [26] threshold for strong evidence, the results of the tangram task tended to support the absence of a sleep effect. These findings do not support those of previous studies that reported a decline in creative ability due to sleep deprivation but are consistent with the findings suggesting that performance on complex tasks may be more resilient to the effects of sleep deprivation. The brain may maintain a certain level of creative function under sleep-deprived conditions, similar to that when fully rested [1, 31]. Furthermore, Sasmita et al. [32] suggested that the attention function could be preserved during sleep deprivation when a reward is involved. As creativity tasks often involve moments of insight and problem-solving, they may engage the brain’s reward system, which could help maintain performance despite declines in subjective and objective alertness. Given that the DAT has been reported to be more enjoyable than other creativity tasks [23], it may be the most robust against the effects of partial sleep deprivation.

Our results suggest that partial sleep deprivation with a 2-h time-in-bed does not impair linguistic idea generation or performance in insight-based problems. This could partly explain why individuals in creative professions who are often sleep-deprived may still function at a highly creative level. However, continuing to work while sleep deprived may come at the cost of other cognitive functions and health issues. Additionally, previous studies have shown that individuals with high creativity are more prone to insomnia and sleep disturbances [33, 34]. Thus, this study provides valuable evidence highlighting the need to raise awareness about the importance of maintaining sleep health among creative professionals.

## Data Availability

The datasets used and/or analyzed in the current study are available from the corresponding author upon reasonable request.

## Abbreviations

ANOVA: Analysis of variance
BFs: Bayes factors
EEG: Electroencephalography
DAT: Divergent Association Task
PSQI: Pittsburgh Sleep Quality Index
PVT: Psychomotor Vigilance Test
REM: Rapid eye movement
SSS: Stanford Sleepiness Scale
STAI: State-Trait Anxiety Inventory
